# Determinants and economic burden of HIV/AIDS in Iran: a prospective study

**DOI:** 10.1186/s12913-023-09229-6

**Published:** 2023-03-14

**Authors:** Tahmineh Reshadat-Hajiabad, Alireza Khajavi, Ali Mohammad Hosseinpour, Amin Bojdy, Amir Hashemi-Meshkini, Mehdi Varmaghani

**Affiliations:** 1 Social Determinants of Health Research Center, Mashhad University of Medical Sciences, Mashhad, Iran; 2 Department of Health Economics and Management Sciences, School of Health, Mashhad University of Medical Sciences, Mashhad, Iran; 3 Iran University of Medical Sciences, Tehran, Iran; 4 Mashhad University of Medical Sciences, Mashhad, Iran; 5 Infections Disease Department, Faculty of Medicine, Mashhad University of Medical Sciences, Mashhad, Iran; 6 Department of Pharmacoeconomics and Pharmaceutical Administration, School of Pharmacy, Tehran University of Medical Sciences, Tehran, Iran

**Keywords:** HIV, AIDS, Cost of illness, Iran

## Abstract

**Background:**

Since the start of the AIDS outbreak, the human immunodeficiency virus (HIV) has infected about 84.2 million people, and approximately 40.1 million people have died due to AIDS-related diseases. So, this study aims to provide a comprehensive population-based description of patient costs and the economic burden of HIV/AIDS in Iran.

**Methods:**

The study population of this cross-sectional cost-of-illness study consisted of HIV-infected patients who were receiving services in Mashhad and were under the supervision of BIDCC. There are four BIDCC centers in Mashhad, we considered all patients referred to these centers. Costs data were evaluated from a social perspective with a bottom-up approach and as a prevalence based. The data from 157 individuals were included in the study. For collecting data on direct and indirect costs belonging to patients and their families, a questionnaire was developed. Also, the Demographic characteristic of participants and the stage of the disease and Transmission category were analyzed.

**Results:**

In this study, 57.32 of the subjects were Male. The majority of participants in this study were in the age group 30–59 years (n = 124,78.98%). Based on where the patients live, the majority of patients have lived in the urban region (n = 144, 91.72%). The most common way to transmit this disease is through unprotected sex (30.57%) and then Infected spouse (28.03%), and then injecting drugs (21.02%). The highest cost of this disease is attributed to medicine (10339.32 $ for 6 months), after medicine, the cost of tests was 9101.22 $.

**Conclusion:**

It seems that to reduce costs for patients with disease HIV/AIDS, the focus should be on diagnostic tests and care. Early diagnosis and rapid initiation of antiviral treatments can be effective in preventing serious and debilitating diseases.

**Supplementary Information:**

The online version contains supplementary material available at 10.1186/s12913-023-09229-6.

## Introduction

Since the start of the AIDS outbreak, the human immunodeficiency virus (HIV) has infected about 84.2 million people, and approximately 40.1 million people have died due to AIDS-related diseases [1]. United Nations program on HIV/AIDS (UNAIDS) has estimated that about 38.4 million people were living with human immunodeficiency virus (HIV) around the world. Also, People newly infected with HIV in 2021 were 1.5 in 2021, and AIDS-related deaths in 2021 were 650,000 [2–4]. Consequently, HIV/AIDS is one of the main burdens of disease worldwide [5].

Regarding the newest estimates of the UNAIDS on HIV/AIDS, a total of 53,000 people (95% CI [38,000–140,000) people are living with HIV (all ages) in Iran until 2021, among them, women aged 15 and over, the men aged 15 and over, and the Children aged 0 to 14 were estimated to be about 17,000 [12,000–44,000], 35,000 [25,000–96,000], and 1,400 [1,000–3,200], respectively. Furthermore, AIDS-related deaths of all ages in Iran in 2019 were 2,500 [1,200–5,600], of which 2,000 deaths were recorded for men and they are between 16 and 40 years old [2, 6].

The long duration of this disease has created the need for patients for long and high-level treatments, which has caused the reduction or loss of savings and household income and increased the debts of the patients’ families and the patients themselves. The high costs of health care for patients and their families make them neglect the ability to prepare nutritious food, invest and work, and even affect their children’s education and many other things. has reduced the death rate, but still every year a large number of infected patients lose their lives due to this disease. (1) The most important economic effect of HIV/AIDS is death at the working age [7]. Losing working time, changing jobs, time spent caring for the sick, and days that cannot go to work due to disability [8]. All these pieces of evidence indicate severe economic pressure for patients with HIV/AIDS who have to pay the high costs of care and try to compensate for lost income, reduced household income and savings, loans, loans, and children being removed from school. The most important cases are the effects of HIV/AIDS on families [9, 10].

The studies on economic burden and cost-of-illness (COI) evaluate the direct and indirect costs of particular diseases in a defined period [11]. Estimating direct and indirect medical costs due to different disease stages is valuable for partial and full economic evaluation studies because it offers a comprehensive insight into affected costs on prevention or screening strategies in non-communicable diseases in society [12]. Precise knowledge of COI enables policymakers and planners to prioritize healthcare plans and policies, and interventions, and even better allocate the resources to healthcare according to budget limitations to achieve policy effectiveness [13].

According to our knowledge, HIV patient costing studies in Iran have not comprehensively evaluated the economic impact, direct and indirect costs of illness, and also productivity costs due to HIV/ AIDS. This study aims to provide a comprehensive population-based description of patient costs and the economic burden of HIV/AIDS in Iran.

## Method

### Study setting

The study was carried out in the northeastern province of Iran (Khorasan Razavi). Mashhad, the capital city of this province, had approximately 3,001,184 population in 2022, a population density of around 8550.38 people per square kilometer [14].

### Patients and methods

**Fig. 1:**
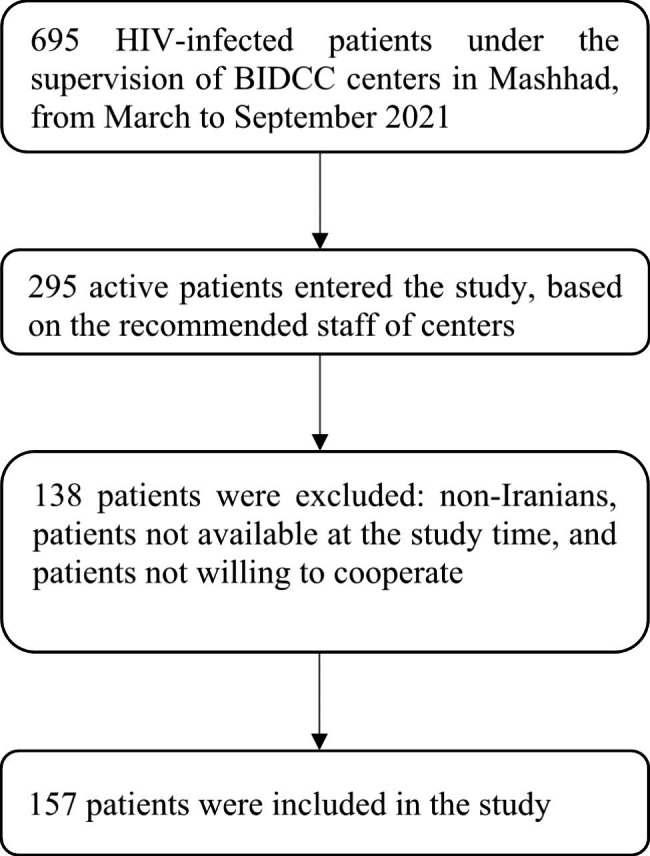
Participant recruitment and follow-up

The study population of this cross-sectional study consisted of HIV-infected patients who were receiving services in Mashhad and were under the supervision of Behavioral and Infectious Diseases Counseling Centers in Mashhad (BIDCC), from March to September 2021. There are four BIDCC centers in Mashhad, we considered all patients referred to these centers. The number of patients identified in this partial economic evaluation and cost analysis in Mashhad at the beginning of the study was 695 patients, which according to the recommended staff of each center, a total of 295 active patients entered our study. Out of this number, non-Iranian patients and patients who were not available at this time, as well as patients who did not want to cooperate, were excluded. Finally, 157 patients were included in the study (Fig. 1). This study was descriptive-analytical, and the perspective of society was used for computing the direct and indirect costs. The calculated expenses contained out-of-pocket costs and government and insurance payments. The monetary unit considered in this study was the US dollar.

### Inclusion and exclusion criteria for selecting of sample

The inclusion criteria included officially registered subjects, who regularly visited health centers to receive services and were willing to participate in the study. Exclusion criteria included those who died during the study period.

### Direct medical costs

The data relating to medical costs including direct medical costs and direct nonmedical costs were gathered prospectively and simultaneously from several paths for better accuracy: through considering medical and financial documents of the patients, face-to-face interviews with subjects, as well as interviews with employees and specialists, who cared for a patient. The leading items of direct medical costs comprised visits by a family physician and specialist (inpatient and outpatient), diagnostic services, laboratory tests, hospital stay, other prescribed drugs, and booster medicines. The average number of every service received for inpatients was taken out from medical records and for outpatients extracted by face-to-face interviews with patients. At the end of retrieved data, the mean number of gathered data for each patient was confirmed by specialists and checked by national tariffs. The mean direct medical costs per patient in HIV disease were defined as follows: a total of three costs per patient was equal (mean number of specialist and general physician visits per three months × visit fee) + (mean number of diagnostic received services per three months × cost for every diagnostic service) + (mean number of tests per three months × cost for every test) + (the cost of each unit of prescribed drugs × the number of medicines on a three month of treatment) + (mean hospital stay per six months × cost per each day) [15].

### Direct nonmedical costs

The information on direct nonmedical costs in patients with HIV diseases was achieved using patients’ self-declaration information by patients using face-to-face or telephone interviews through a checklist that was prepared in advance to calculate costs. Direct non-medical costs include transportation (inner-city and long-distance), food and accommodation for the patient and their companions, purchase of any medical supplies and aids in the treatment process (such as wheelchairs, walkers, home care beds), and changes in their home due to illness (for example, the installation of an elevator for a person who is paralyzed following HIV / AIDS). In this study, because there was no cost to patients in other areas, only the cost of the patient’s travel was considered [16].

### Indirect costs

Indirect costs retrieve from reduced productivity of patients or family members in response to illness, death, or treatment. Productivity loss cost is due to the absence from work of patients and their families, who care for them. In total, the following items were calculated as productivity lost in this study:

- Number of days because of disability of the patient and companions including time spent receiving outpatient and inpatient services such as travel time, days of hospitalization, number of days of recovery after discharge.
- Job loss as a result of illness.
- Number of days spent in the hospital and nursing at home, number of days of disability of family, relatives, and friends due to patient care.
- The rate (percentage) of decrease in patient income due to illness.

In the present study, the human capital approach based on the minimum wage has been used to calculate indirect costs [17]. The data required to calculate this part of the costs are also obtained based on patients’ self-report through face-to-face or telephone interviews with patients and patient companions.

Finally, the indirect cost of each person and the disease status was calculated using the following formula:

Minimum daily wage * Total number of days of disability of patient and companions = indirect cost.

The average national wage for laborers, who worked in Iran was calculated as 885,165 Iranian Rial (USD 1 = IRR 279,199 [Iranian Rial]) per day, and this number was multiplied by the number of lost days. Minimum monthly wage (26554950 Rial = 95.11 $) Minimum daily wage (88,165 Rial = 3.17 $).

## Results

### Determinants of AIDS

In this study, a total of 157 AIDS patients were involved based on inclusion criteria. 57.32 of the subjects were Male. The majority of participants in this study were in the age group 30–59 years (n = 124,78.98%). Based on where the patients live, the majority of patients have lived in the urban region (n = 144, 91.72%). more than 15% of patients had to change their employment status. All the socioeconomic and descriptive statistics of the studied subjects were demonstrated in Table 1.

**Table 1:** Frequency of some demographic characteristics (based on sex) of patients participating in the study

Demographic characteristics		Male number(percentage)	Female number (percentage)
		90(57.32)	67 (42.68)
Age	Under 5 years	0	2 (2.99)
	5 to 17 years	5 (5.56)	6 (8.96)
	18 to 29 years	2 (2.22)	6 (8.96)
	30 to 59 years	75 (83.33)	49 (73.13)
	upper 60 years	8 (8.89)	4 (5.97)
Place of residence of the patient in terms of urban/rural	Urban	85(94.44)	59(88.06)
	Rural	5(5.56)	8(11.94)
Marital status	married	44(48.89)	40(59.70)
	Divorced	22(24.44)	6(8.96)
	deceased wife	4(4.44)	13(19.44)
	Single	20(20.22)	8(11.94)
Education	illiterate	3(3.33)	9(13.43)
	High school	61(67.78)	41(61.19)
	diploma	23(25.56)	15((22.39)
	College education	3(3.33)	2(2.99)
Job change due to disease	yes	16(17.78)	8(11.94)
	no	74(82.22)	59(88.06)
Unemployment due to HIV	yes	14(15.56)	8(11.94)
	no	76(84.44)	59(88.06)
Having insurance	yes	70(77.78)	59(88.06)
	no	20(22.22)	8(11.94)
Insurance type	no insurance	20(22.22)	8(11.94)
	Iranian health insurance	48(53.33)	41(61.19)
	social security insurance	20(22.22)	17(25.37)
	Armed forces insurance	2(2.22)	1(1.49)
Supplementary insurance	yes	8(8.89)	4(5.97)
	no	82(91.11)	63(94.03)
Head of Household	yes	62(68.89)	14(20.90)
	no	28(31.11)	53(79.10)
Fixed place to live	yes	87(96.67)	64(95.52)
	no	3(3.33)	3(4.48)

The majority of patients were in stage 1 HIV diseases (n = 135, 85.99%). This amount in men and women is equal to n = 76, 84.44%, and 59,88.06%, respectively (Table 2). According to the results of the study, the most common way to transmit this disease is through unprotected sex (30.57%) then an infected spouse (28.03%), and then injecting drugs (21.02%). So more than 40% of the studied men contracted the disease through sexual intercourse. Among women, the most common cause of infection was through an infected spouse (Table 3).

**Table 2:** Frequency of some demographic characteristics (based on gender) of patients participating in the study (stages of the disease)

Characteristics		Male	Female
		number (percentage)	number (percentage)
		90(57.32)	67 (42.68)
Stage of the disease	1	76(84.44)	59(88.06)
	2	6(6.67)	4(5.97)
	3	6(6.67)	4(5.97)
	4	2(2.22)	0

**Table 3:** Frequency of some demographic characteristics (based on gender) of patients participating in the study (transmission route)

Characteristics		Male	Female
		number (percentage)	number (percentage)
		90(57.32)	67 (42.68)
Transmission category	Injecting drug use	32(35.56)	1(1.49)
	Unsafe sex	40(44.44)	8(11.94)
	Transmission through an infected spouse	3(3.33)	41(61.19)
	Transmission from Mother-to-child	6(6.67)	9(13.43)
	Transmitted through blood	2(2.22)	3(4.48)
	Transmission through dental visits	1(1.11)	0
	Transmission through tattoos	0	1(1.49)
	Unknown	6(6.67)	4(5.97)

The results show that 41.4% of the patients in the study have an addiction history. Among the patients who had an addiction, about 26.75% had a history of injecting drugs, and among those who had a history of injection, 19.75% had a history of joint injection. Also, 36.94% of the patients had a history of prison, 44.59% of the patients had a history of unprotected sex, and 27.39% had multiple sexual partners (Table 4).

**Table 4:** Frequency of some demographic characteristics (based on gender) of patients participating in the study (specific records)

Characteristics		male	female
		number (percentage)	number (percentage)
		90(57.32)	67 (42.68)
Having a history of addiction	yes	60(66.67)	5(7.46)
	no	30(33.33)	62(92.54)
Having a history of injection	yes	39(43.33)	3(4.48)
	no	51(56.67)	64(95.52)
Having a history of joint injection	yes	29(32.22)	2(2.99)
	no	61(67.78)	65(97.01)
Having a prison record	yes	54(60.00)	4(5.97)
	no	36(40.00)	63(94.03)
Having unsafe sex	yes	57(63.33)	13(19.40)
	no	33(36.67)	54(80.60)
History of sexual relations with non-spouse	yes	55(61.11)	12(17.91)
	no	35(38.89)	55(82.09)
Having multiple sexual partners	yes	35(38.89)	8(11.94)
	no	55(61.11)	59(88.06)
History of venereal disease	yes	8(8.89)	2(2.99)
	no	82(91.11)	65(97.01)

### Cost

Considering that the government covers almost all the necessary treatment needs of HIV/AIDS patients, we have divided the costs into two parts: the patient’s share and the government’s share, and calculated each one separately. In general, the highest cost of this disease is attributed to medicine (10339.32 $ for 6 months), and of this cost, 6988.67 $ was the government’s share, and 3350.65 $ was the patient’s share. After medicine, the cost of tests was 9101.22 $, of which 8464.61 $ was the government’s share and 636.6 $ was the patients’ share. Among the expenses of the patients, hospitalization and dental costs were 1485.67 $ and 770.52 $, respectively for all patients in 6 months (Table 5).

**Table 5:** Direct medical costs* (During 6 months)

Type of service	Average direct medical costs per patient)patient share(	Average direct medical costs per patient )Government Share(	Total direct medical costs )Patient share(	Total direct medical costs )Government share(	Total direct medical costs )Patient and government costs(
Specialist visit	2.07 ± 3.58	3.02 ± 1.17	303.95	471.37	775.33
Medicine	25.46 ± 53.27	44.51 ± 15.15	3350.65	6988.67	10339.32
Psychologist	0	9.18 ± 0.74	0	1193.77	1193.77
Midwife visit	0.12 ± 0.43	1.07 ± 0.57	3.58	30.21	33.79
Experiments	5.41 ± 13.34	60.89 ± 55.69	636.60	8464.61	9101.22
Dental	62.52 ± 77.87	0	770.52	0	770.52
Emergency services	5.65 ± 4.75	0	130.01	0	130.01
Radiology	18.68 ± 22.48	0	276.68	0	276.68
Sonography	5.92 ± 6.26	0	44.37	0	44.37
Physiotherapy	132.88 ± 159.69	0	398.64	0	398.64
Hospitalization	116.48 ± 147.90	5.51 ± 19.86	1485.67	71.63	1557.31
Chemotherapy	130.73 ± 0	0	130.73	0	130.73
Condom	1.59 ± 2.62	4.18 ± 4.07	130.98	355.66	486.64
Methadone	8.43 ± 0.82	46.41 ± 5.08	210.60	1253.08	1463.68

*The monetary unit considered in this study was the US dollar

In Table 6, the average cost per patient and the cost of the total days lost due to HIV/AIDS are calculated separately for accompanying patients and caregivers, and the largest share of this cost is for the patient (1696.9 ± 848.28 and in general, the cost of lost days for all patients in these 6 months is 12794.56 $, the largest share of which is related to the patients themselves with a cost of about 11583.48 $.

**Table 6:** The average cost* per patient and the cost of the total days lost due to HIV/AIDS

Type of service	Total number of days lost	Average per patient(day)	Average cost lost during 6 months per patient	Total cost lost during 6 months
Days lost due to illness (patient)	4989	31.7	848.28 ± 1696.90	11583.48
Days lost due to illness (accompanying the patient)	48	0.3	8.40 ± 27.07	57.06
Days lost due to illness (Caring for the patient)	371	2.3	37.23 ± 310.34	1154.01
Total	5408	34.4	745.49	12794.56

*The monetary unit considered in this study was the US dollar

Table 7 also shows the direct and indirect medical costs as well as the indirect costs due to the lost productivity of the patients separately between the patients with addiction and the patients without addiction, as we can see the obvious difference between the patients with a history there is addiction and no history of addiction.

**Table 7:** Direct medical costs and Indirect costs* by patients with a history of addiction and without a history of addiction

Type of service	Addiction	No addiction
Direct medical costs(Patient share)	65.05 ± 115.83	51.31 ± 106.03
Direct medical costs(Government share)	129.57 ± 70.6	113.11 ± 55.44
Direct nonmedical costs(Patient share)	23.07 ± 67.50	31.65 ± 44.30
Direct nonmedical costs(Government share)	0	0
Days lost due to illness(patient)	1313.33 ± 2039.29	519.72 ± 1322.05
Days lost due to illness (accompanying the patient)	1.70 ± 7.09	13.14 ± 34.14
Days lost due to illness (Caring for the patient)	41.65 ± 287.18	34.11 ± 327.22
The percentage of attracting income to HIV AIDS	0.2 ± 0.33	0.19 ± 0.3
The percentage of income reduction due to HIV AIDS	0.44 ± 0.42	0.18 ± 0.33

*The monetary unit considered in this study was the US dollar

## Discussion

This study was the first comprehensive study that has considered the economic burden and Determinants of HIV/AIDS on patients in Iran with a prospective approach from a societal perspective. In this study, the costs are separated into two parts: the patient’s share and the government’s share. The reason for this is that since HIV/AIDS is a special and contagious disease, the costs related to the medical services of patients, including tests, doctor’s visits, medicine, consultation, etc. are offered to patients for free, are all over the world. On the other hand, due to the specific conditions of the disease and the effect that this disease has on all the organs of the body, as well as the weakness of the immune system due to this disease and the need to strengthen the body, patients also pay expenses out of their pockets for getting the additional therapy. In this study, an attempt has been made to calculate all these costs. During these six months, it was estimated that 39844.7 $ of direct and indirect costs for HIV/AIDS disease for 157 patients participating in the research, that the government spent 18829.03 $ for participating patients with HIV/AIDS, and the patients also spent 8221.1 $ have paid from their own pockets for direct medical and non-medical expenses, which is 21015.66 $ for the patient, if we have included the days lost due to disability due to illness.

Average total productivity losses (Days lost due to illness of patients, accompanying the patient, and Caring for the patient) because of HIV/AIDS was to be high in a monthly period (5.73 days). The findings of this study are in line with the findings of the Nepal study so in their study Average total productivity losses were found 5.05 days [18]. In our study, the proportion of average total productivity losses to total costs (sum direct cost and indirect cost and productivity loss) of this study was 31.1%. while in the Nepal study average, total productivity losses to total costs were found 32.3% [18].

According to the results, there was the cost per patient varied based on disease stage. Patients in stage one incurred the major cost for society and the health system and stage four incurred the minimum cost. Also, the highest cost is the direct cost related to antiviral drugs. Based on the current study findings, in general10339.32$ were spent on drugs by the government and patients, of which 6988.67$ were paid by the government. And 3350.65$ have been spent by the patients. The average cost of antiviral drugs per patient is estimated to be approximately 25.46 ± 53.27 and 44.51 ± 15.15 $, respectively, for the patient’s share and the government’s share. Even though no related study has been carried out on this disease in patients with HIV in Iran, the results of this study are consistent with the results of other studies in this field. A study conducted by Julien Kuhlmann and colleagues in Bogota, Colombia in 2017, showed that the cost of providing medicine is the largest for HIV/AIDS. In this study, the drug cost for each patient was estimated at an average of 8616 US dollars, which includes 75% of the total costs [19]. Also, our study is consistent with the results of a study conducted by Sarah Mostardt et al. The results of this study showed that the cost of antiviral drugs is the most expensive in HIV + disease [20]. Generally, the results of other studies in North America and Europe are consistent with our results based on the percentage of spending on drugs [20–22]. Poudel et el. in their study found that the highest cost of the direct cost was related to the Cost of diagnosis or test, so their results were not in line with the results of our study [18]. Briefly, it seems that there are two reasons for the high cost of medicines in HIV/AIDS patients: First, the complaint of most patients with this disease is the breakdown of the immune system, which forces patients to use strengthening medicines that these medicines constitute a large part of patients’ expenses. Second, in addition to the HIV/AIDS disease itself, these patients gradually suffer from a variety of chronic diseases, which may not happen in healthy people or may occur in old age. For patients with HIV/AID, the same issue brings the cost of medicine for all kinds of diseases, which is also expensive, and in general, it is because the cost of medicine is higher than other services.

Because many patients with HIV, are obliged to visit health care centers far from their place of dwelling to have necessary treatment, the direct and indirect nonmedical costs imposed on the patient and the government are increased. Furthermore, the indirect costs of getting treatment for these patients are very high, because many patients are forced to request long-term leave or dismissal from their workplace due to social issues. So, in this study results demonstrated that per each patient, there were 31.7 days away from work. According to the results of this study, a large percentage of patients are of working age and are less than 60 years old, so, this disease imposes a large cost on society due to lost productivity.

We had some limitations in this study. First, in cost estimation, efforts have been made to calculate different types of costs such as direct treatment and non-treatment costs as well as indirect costs. But intangible costs, including pain, suffering, stress and anxiety, and social stigma of patients and those around them, especially in the severe stages of the disease, which exist to a significant extent, have not been included in the calculations. However, due to the lack of accurate and appropriate calculation methods, such costs are usually not included in economic burden studies. The second weakness is that we could not include a large number of patients in the study due to a lack of access and lack of cooperation.

Because a large number of patients participating in the study had a history of addiction, a comparison was made between patients with and without a history of addiction in this study, which showed that patients with a history of addiction incurred more costs. Also, the comparison of the cost of lost days due to illness showed that the number of days lost by patients who had an addiction and their companions and caregivers is far more than those who do not have a history of addiction.

The strength of the present study was using the bottom-up approach, in which the researchers could have collected accurate information on the direct and indirect costs of HIV patients. The fact that the cost information was collected natively and through the self-declaration of patients and specialists by the researcher from the centers under study was based on observation, and can be another positive point in the current research. In the end, despite all the difficulties of accessing these patients and building trust in them, this research happened for the first time in Iran. The limitations of the present study was the sample consisted of HIV-infected patients under the supervision of BIDCC centers. As a probable source of bias, the sample might underrepresent the newly-diagnosed cases and subjects of high socioeconomic status, due to the social stigma in a religious city such as Mashhad.

## Conclusion

The results of the present study showed that HIV/AIDS can be considered a disease that imposes a large economic burden on the government and patients from society’s point of view. It involves society, and in addition to the treatment issues, it can affect all the economic and social issues of society, therefore, as many measures as possible for the prevention and timely diagnosis of this disease should be put on the agenda of the government and the policymakers should prioritize the health system of HIV/AIDS. Furthermore, it seems that to reduce costs for patients with disease HIV/AIDS, the focus should be on diagnostic tests and care. Early diagnosis and rapid initiation of antiviral treatments can be effective in preventing serious and debilitating diseases. The costs of early diagnosis and then early treatment are far less than the costs of treatment and reduced productivity. Having special insurance for these patients can greatly reduce the out-of-pocket payments of these patients. To reduce costs, we should pay attention to treatment available to all patients, provide services at the regional level, and set up more comprehensive service centers for the care of HIV/AIDS patients. We can consider private subsidies for patients and public hospitals. prepare for low-cost diagnostic tests, especially for people who live on the outskirts of cities and pregnant women at the time of delivery. These policies not only reduce the economic burden of the disease but also encourage patients to test and start treatment. A complete policy for livelihood support of patients can be skill development and income generation programs.

## Electronic supplementary material

Below is the link to the electronic supplementary material.

Supplementary Material 1. The data on the Determinants and economic burden of HIV/AIDS in Iran in 2022

## Data Availability

The datasets used and/or analyzed during the current study are available from the corresponding author upon reasonable request.

## Abbreviations

MUMS: Mashhad University of Medical Sciences
GP: General Practitioners
